# Long-Term Impact of the COVID-19 Pandemic on In-Hospital Antibiotic Consumption and Antibiotic Resistance: A Time Series Analysis (2015–2021)

**DOI:** 10.3390/antibiotics11060826

**Published:** 2022-06-20

**Authors:** Marianna Meschiari, Lorenzo Onorato, Erica Bacca, Gabriella Orlando, Marianna Menozzi, Erica Franceschini, Andrea Bedini, Adriana Cervo, Antonella Santoro, Mario Sarti, Claudia Venturelli, Emanuela Biagioni, Irene Coloretti, Stefano Busani, Massimo Girardis, José-María Lòpez-Lozano, Cristina Mussini

**Affiliations:** 1 Department of Infectious Diseases, Azienda Ospedaliero-Universitaria of Modena, 41124 Modena, Italy; mariannameschiari1209@gmail.com (M.M.); gabriella.orlando7@virgilio.it (G.O.); marymenozzi@gmail.com (M.M.); ericafranceschini0901@gmail.com (E.F.); andreabedini@yahoo.com (A.B.); adriana.cervo@gmail.com (A.C.); antonella.santoro7@gmail.com (A.S.); 2 Infectious Diseases Unit, Department of Mental Health and Public Medicine, University of Campania Luigi Vanvitelli, 80138 Naples, Italy; lorenzoonorato@libero.it; 3 Clinic of Infectious Diseases, Department of Infectious Diseases, University of Modena, 41124 Modena, Italy; cristina.mussini@unimore.it; 4 Clinical Microbiology Laboratory, Azienda Ospedaliero-Universitaria of Modena, 41124 Modena, Italy; sarti.mario@aou.mo.it (M.S.); venturelli.claudia@policlinico.mo.it (C.V.); 5 Intensive Care Unit, Azienda Ospedaliero-Universitaria of Modena, 41124 Modena, Italy; emanuela.biagioni@gmail.com (E.B.); irenecoloretti@gmail.com (I.C.); stefano.busani@unimore.it (S.B.); massimo.girardis@unimore.it (M.G.); 6 Medicine Preventive-Infection Control Team, Hospital Vega Baja, 03314 Orihuela-Alicante, Spain; jmloploz@gmail.com

**Keywords:** COVID-19, infection control, antimicrobial stewardship, antimicrobial resistance, MDROs, *Acinetobacter baumannii*, *Pseudomonas aeruginosa*, *Staphylococcus aureus*, *Escherichia coli*, *Clostridioides difficile*

## Abstract

The coronavirus disease 2019 (COVID-19)-pandemic-related overload of health systems has compromised the application of antimicrobial stewardship (AS) models and infection prevention and control (IPC) programs. We aimed to evaluate the impact of COVID-19 on antimicrobial consumption (AC) and antimicrobial resistance (AMR) in the University Hospital of Modena. A time series analysis with an autoregressive integrated moving average model was conducted from January 2015 to October 2021 to evaluate the AC in the whole hospital and the intensive care unit (ICU), the incidence density (ID) of bloodstream infections (BSIs) due to the main multidrug-resistant organisms, and of *C. difficile* infections (CDIs). After an initial peak during the COVID-19 period, a decrease in the trend of AC was observed, both at the hospital (CT: −1.104, *p* = 0.025) and ICU levels (CT: −4.47, *p* = 0.047), with no significant difference in the single classes. Among the Gram-negative isolates, we observed a significant increase only in the level of BSIs due to carbapenem-susceptible *Pseudomonas aeruginosa* (CL: 1.477, 95% CI 0.130 to 2.824, *p* = 0.032). Considering Gram-positive bacteria, an increase in the level of BSIs due to methicillin-resistant Staphylococcus aureus and in the trend of CDIs were observed, though they did not reach statistical significance (CL: 0.72, 95% CI −0.039 to 1.48, *p* = 0.062; CT: 1.43, 95% CI −0.002 to 2.863, *p* = 0.051; respectively). Our findings demonstrated that the increases in AMR and AC that appeared in the first COVID-19 wave may be later controlled by restoring IPC and AS programs to pre-epidemic levels. A coordinated healthcare effort is necessary to address the longer-term impact of COVID-19 on AC to avoid irreversible consequences on AMR.

## 1. Introduction

After almost two years of the coronavirus disease 2019 (COVID-19) pandemic, every healthcare system has implemented profound changes to prevent the spread of severe acute respiratory syndrome coronavirus 2 (SARS-CoV-2), which may have had a multi-faceted impact on antibiotic resistance. 

The global SARS-CoV-2 crisis has led to healthcare personnel primarily committing to the management of COVID-19, which has affected the correct application of antimicrobial stewardship (AS) models and the proper implementation of infection prevention and control programs (IPCs). Improved isolation practices, maximal use of personal protective equipment (PPE), and adherence to hand hygiene have been beneficial. Still, the focus may have shifted from patient-to-patient contact precautions to personal protection from SARS-CoV-2 exposure; therefore, the risk of cross-transmission became more significant. These attitudes fostered hospital outbreaks and worked as multidrug-resistant organism (MDRO) amplifiers during the COVID-19 epidemic [1].

Although a relatively low rate of bacterial infection among hospitalized COVID-19 patients was reported, ranging from 5 to 27%, broad-spectrum antibiotics have commonly been prescribed, both for prophylaxis and treatment [2]. A recent systematic review and meta-analysis estimated a prevalence of co-infection in only 3.5% of patients (95% CI 0.4–6.7%) and secondary bacterial infection in 14.3% of patients (95% CI 9.6–18.9%) [3]. Similarly, a multicenter prospective UK cohort reported a low incidence of microbiologically confirmed bacterial infections, mainly secondary infections, in COVID-19 patients and frequent use (up to 85.2%) of broad-spectrum antimicrobials [4]. These low rates of infection are in contrast with the high levels of antimicrobial prescriptions, with 70% of COVID-19 patients receiving at least one antibiotic course during their hospital stay, increasing to up to 80–100% for critically ill COVID-19 patients [2,5,6,7,8]. The wide use of broad-spectrum empirical antibiotic therapy was warranted by the exponential rise in ICU admissions, together with invasive procedures and the consequent increase in nosocomial infections. According to other colleagues, not only did patients admitted to ICUs have bacterial coinfection or superinfections more often than COVID-19 patients admitted to ordinary wards [9] but these infections were more often due to MDROs [8].

Higher rates of healthcare-associated infections (HAIs) during the COVID-19 pandemic were reported from several sources [10,11,12,13], in particular, ventilator-associated pneumonia (VAP) [14]. Nevertheless, the exact prevalence of MDROs among COVID-19 patients was not accurately estimated. As in previous epidemic experiences with SARS-CoV, a significant increase in the rate of methicillin-resistant *Staphylococcus aureus* (MRSA) was observed during the SARS-CoV-2 pandemic [15]. Grasselli et al., who analyzed the most comprehensive cohort of critically ill patients with COVID-19 in Italy, pointed out the significant risk of HAIs in COVID-19 patients resulting from MDROs; indeed, 35% of the 759 HAIs analyzed were due to MDROs [12]. The latest WHO and ECDC surveillance of antimicrobial resistance (AMR) in Europe, which referred to 2020 figures and was based on Central Asian and European Surveillance of Antimicrobial Resistance (CAESAR) and the European Antimicrobial Resistance Surveillance Network (EARS-Net), reported a high level of resistance to third-generation cephalosporins (3GCs) and carbapenems in *Klebsiella pneumoniae*, as well as higher rates of typical healthcare-associated pathogens, such as carbapenem resistance in *Acinetobacter* spp. and *Enterococcus faecium*. At the same time, fewer *Streptococcus pneumoniae* isolates were reported in 2020 than in previous years, possibly due to the reduced circulation of respiratory pathogens in the community during the lockdowns or the application of physical measures to control the spread of SARS-CoV-2 [16].

Despite the extreme variability between regions, these data suggest a decreasing trend of co-infections due to community pathogens in contrast with an excess of MDROs responsible for COVID-19 superinfections that are probably related to increased relative rates of hospital-onset pathogen clusters [17,18,19,20,21].

More outstanding efforts to improve data-driven research and surveillance in hospitals during the COVID-19 pandemic are urgently needed to reinforce target IPC and AS programs, which have been severely relaxed during the pandemic. Our aim was to provide a precise evaluation of COVID-19’s impact on antimicrobial consumption and AMR after two years of the pandemic.

## 2. Materials and Methods

### 2.1. Setting

The University Hospital of Modena is a tertiary care hospital in northern Italy, with approximately 700 beds, an average of 30,000 admission per year, 200,000 hospitalization days, and a yearly occupancy of more than 90%. 

Since 2011, an ICP was progressively implemented, which included an active surveillance system that involved the microbiology laboratory and all infection control staff promptly identifying all patients colonized or infected with carbapenem-resistant Gram-negative bacteria (CR-GNB). A hospital-wide rectal screening for all CR-GNB was performed at admission and repeated weekly. In addition, all CR-GNB-colonized patients are cared for with contact precautions, using gowns and gloves for any patient contact. As of 2012, a multimodal hand hygiene project was implemented according to the WHO recommendations [22]. An antimicrobial stewardship program, in addition to standard consultations, started in September 2014, with prospective audit and feedback (PAF) events performed three times per week by an infectious disease specialist, and a restricted formulary for carbapenems, fluoroquinolones, colistin, and tigecycline was put in place. In 2017, a computerized surveillance system for monitoring antibiotic consumption was introduced, providing real-time data on antibiotic use that is expressed as the defined daily dose (DDD) per 100 patient-days (PD).

On 29th February 2020, the University Hospital of Modena was selected as the “COVID hospital” designated to receive the most significant number of patients affected by SARS-CoV-2 pneumonia in the province, admitting 4164 patients to date with a diagnosis of SARS-CoV-2 infection confirmed via PCR testing on a nasopharyngeal swab. Neither AS nor IC programs were interrupted, but they were both relatively compromised during the epidemic waves. 

### 2.2. Data Collection and Outcomes

The incidences of all bloodstream infections (BSIs), expressed as monthly isolates/100PD for one per person per month/admission (only isolated strains with an interval of 30 days between the previous strain and the next were considered non-duplicates, regardless of phenotype), were considered, including *A. baumannii* (both totally sensitive and carbapenem-resistant), *K. penumoniae* (both totally sensitive and carbapenem-resistant), *P. aeruginosa* (both totally sensitive and carbapenem-resistant), *S. aureus* (both methicillin-sensitive and methicillin-resistant), *E. coli* (both totally sensitive and extended-spectrum beta-lactamases producers), and *E. faecium* (both vancomycin-sensitive and vancomycin-resistant). The incidence of *C. difficile* (CD) was expressed as the monthly total number of CD/100PD. The total number of positive CD samples/tests per laboratory was collected independently of the type of diagnostic test currently used (a positive laboratory assay for CD toxin A and/or B in stools or a toxin-producing *C. difficile* organism detected in stool via culture or other means, e.g., a positive PCR) and without distinguishing between nosocomial and community samples.

The monthly data of antimicrobial consumption were expressed as DDD/100PD, total consumption, and single-class consumptions, both for the whole hospital and the ICU.

The primary outcome was to evaluate the changes in antimicrobial consumption and AMR in the new epidemiological scenario caused by SARS-CoV-2.

The pre-COVID-19 period was defined as January 2015 to February 2020; the post-COVID-19 period included the months from March 2020 to November 2021.

## 3. Results

### 3.1. Antibiotics Consumption

**Figure 1:**
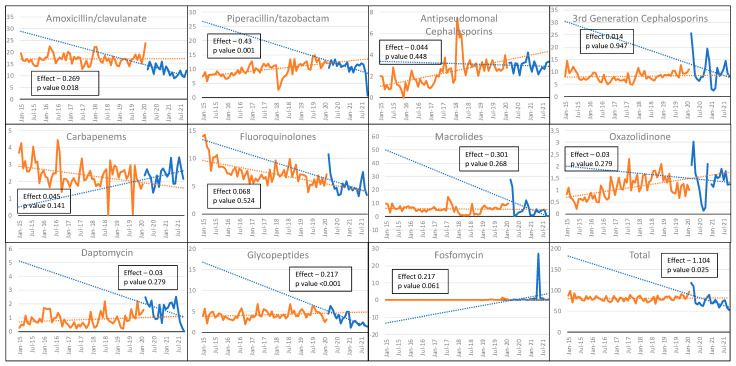
Changes in trends and changes in levels of antibiotic consumption during the pre-COVID-19 (in orange) and COVID-19 (in blue) periods at the whole-hospital level. On the *x*-axis, antibiotic use is expressed as the defined daily dose per 100 patient-days; the *y*-axis represents the time. The total hospital antibiotic consumption is shown in the bottom-right graph.

A total of 987,306 and 314,575 patient days were analyzed during the pre-COVID-19 and COVID-19 periods, respectively. After an initial peak, a decrease in the trend of the overall antibiotic consumption in the whole hospital was observed during the COVID-19 period, with a change in level (CL) of −3.028 (95% CI from −14.72 to 8.67, *p* = 0.025) and a change in trend (CT) of −1.104 (95% CI −2.06 to −0.14, *p* = 0.025); a similar decrease in the trend of consumption was observed for amoxicillin-clavulanate (CT: −0.27, 95% CI −0.49 to −0.05, *p* = 0.018), piperacillin/tazobactam (CT: −0.43, 95% CI −0.69 to −0.17, *p* = 0.001), and glycopeptides (CT: −0.22, 95% CI −0.31 to −0.12, *p* < 0.001). No significant difference was reported in the antimicrobial use of antipseudomonal cephalosporins (CT: −0.44, 95% CI −0.16 to 0.07, *p* = 0.45), 3GCs (CT: 0.014, 95% CI −0.42 to 0.45, *p* = 0.95), carbapenems (CT: 0.045, 95% CI −0.02 to 0.105, *p* = 0.14), fluoroquinolones (CT: 0.068, 95% CI −0.14 to 0.28, *p* = 0.52), macrolides (CT: −0.30, 95% CI −0.84 to 0.24, *p* = 0.27), oxazolidinone (CT: −0.03, 95% CI −0.08 to 0.03, *p* = 0.28), daptomycin (CT: −0.03, 95% CI −0.09 to 0.03, *p* = 0.28), and fosfomycin (CT: 0.28, 95% CI −0.01 to 0.44, *p* = 0.06) (Figure 1 and Supplementary Table S1).

**Figure 2:**
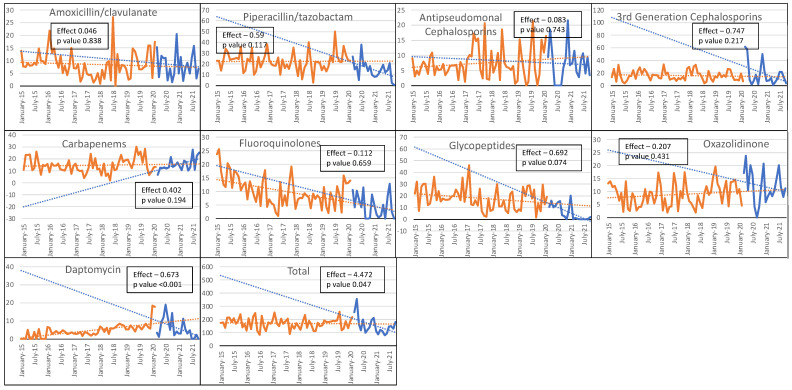
Changes in trends and changes in levels in the antibiotic consumption during the pre-COVID-19 (in orange) and COVID-19 (in blue) periods at the ICU hospital level. On the *x*-axis, antibiotic use is expressed as the defined daily dose per 100 patient-days: the *y*-axis represents the time. The total ICU antibiotic consumption is shown in the bottom-right graph.

Limiting the analysis to the ICUs, a decrease in the trend of all antibiotic use was registered during the COVID-19 period, with a CL of 17.27 (95% CI −35.46 to 70.00, *p* = 0.516) and a CT of −4.47 (95% CI −8.88 to −0.06, *p* = 0.047). Similarly, we observed a reducing trend in the consumption of anti-MRSA agents, such as daptomycin (CT: −0.67, 95% CI −0.98 to −0.37, *p* <0.001) and glycopeptides (CT: −0.69, 95% CI −1.45 to 0.07, *p* = 0.07), although the latter did not reach statistical significance. No significant difference was found for amoxicillin-clavulanate (CT: 0.046, 95% CI −0.39 to 0.49, *p* = 0.838), piperacillin/tazobactam (CT: −0.59, 95% CI −1.33 to 0.15, *p* = 0.117), antipseudomonal cephalosporins (CT: −0.083, 95% CI −0.59 to 0.42, *p* = 0.74), 3GCs (CT: −0.747, 95% CI −1.94 to 0.45, *p* = 0.22), carbapenems (CT: 0.402, 95% CI −0.21 to 1.02, *p* = 0.19), fluoroquinolones (CT: −0.112, 95% CI −0.61 to 0.39, *p* = 0.66), and oxazolidinone (CT: −0.207, 95% CI −0.73 to 0.31, *p* = 0.43) (Figure 2 and Supplementary Table S2).

### 3.2. Bloodstream Infections and CDIs

Regarding the microbiological outcomes, we did not observe a significant variation in the incidence density of BSIs for most of the pathogens evaluated. Interestingly, there was an increase only in BSIs due to carbapenem-susceptible *Pseudomonas aeruginosa* (CL: 1.477, 95% CI 0.130 to 2.824, *p* = 0.032) and in the trend of MRSA, even if they were not statistically significant (CT: −0.078, 95% CI −0.150 to −0.006, *p* = 0.034; CL: 0.722, 95% CI −0.039 to 1.482, *p* = 0.062).

No significant difference was found for both 3GC-susceptible (CT: 0.162, 95% CI −0.137 to 0.461, *p* = 0.284) and 3GC-resistant (CT: 0.036, 95% CI −0.097 to 0.169, *p* = 0.592) Escherichia coli, as well as both carbapenem-susceptible (CT: −0.022, 95% CI −0.169 to 0.125, *p* = 0.767) and carbapenem-resistant (CT: −0.023, 95% CI −0.071 to 0.025, *p* = 0.342) Klebsiella pneumoniae. Regarding non-fermenting Gram-negative organisms, we registered an increase in the incidence level of BSIs due to carbapenem-susceptible P. aeruginosa (CL: 1.477, 95% CI 0.130 to 2.824, *p* = 0.032), while no difference was observed for carbapenem-resistant strains (CT: −0.009, 95% CI −0.051 to 0.033, *p* = 0.675); finally, no significant difference was found for both carbapenem-susceptible (CT: 0.009, 95% CI −0.013 to 0.031, *p* = 0.406) and carbapenem-resistant (CT: 0.020, 95% CI −0.026 to 0.066, *p* = 0.386) Acinetobacter baumanni. Concerning Gram-positive bacteria, after an initial peak, a decreasing trend was observed for BSIs due to methicillin-resistant Staphylococcus aureus (CT: −0.078, 95% CI −0.149 to −0.006, *p* = 0.034), with the increase in level not reaching statistical significance (CL: 0.72, 95% CI −0.039 to 1.48, *p* = 0.062), while no significant variation was observed for methicillin-susceptible isolates (CT: −0.017, 95% CI −0.184 to 0.150, *p* = 0.841). Similarly, no significant difference was found for both vancomycin-susceptible (CT: −0.062, 95% CI −0.180 to 0.057, *p* = 0.269) and vancomycin-resistant (CT: 0.007, 95% CI −0.053 to 0.066, *p* = 0.803) *E. faecium*.

**Figure 3:**
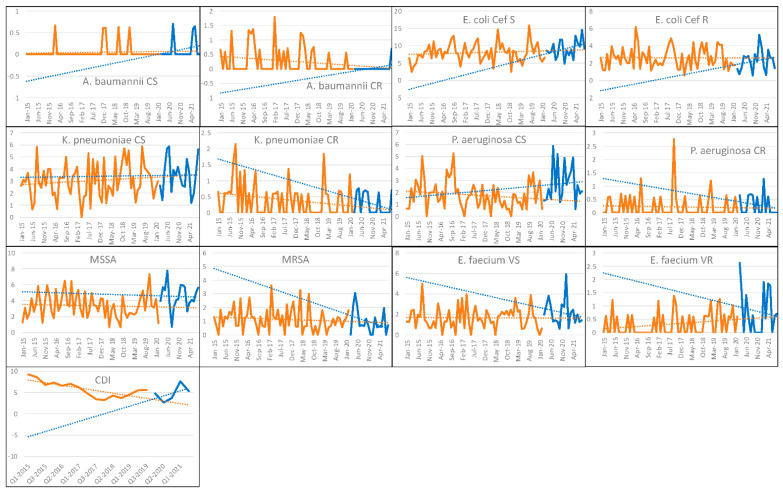
Changes in trends and changes in levels in the incidence density of bloodstream infections and *Clostridioides difficile* infections during the pre-COVID-19 (in orange) and COVID-19 (in blue) periods at the whole-hospital level. On the *x*-axis, antibiotic use is expressed as events per 10,000 patient-days; the *y*-axis represents the time. Legend: CS—carbapenem-susceptible; CR—carbapenem-resistant; Cef S—third-generation cephalosporin-susceptible; Cef R—third-generation cephalosporin-resistant; MSSA—methicillin-susceptible *S. aureus*; MRSA—methicillin-resistant *S. aureus*; *E. faecium* VS—vancomycin-susceptible *E. faecium*; *E. faecium* VR—vancomycin-resistant *E. faecium*; CDI—*Clostridioides difficile* infection.

Finally, an increase in the trend was reported for the incidence of Clostridioides difficile infections, though this did not reach statistical significance (CT: 1.43, 95% CI −0.002 to 2.863, *p* = 0.051). We report an incidence of CDIs at the beginning of the first pandemic wave of 4.787 events per 10,000 patient-days (events/10,000PDs), with a peak of 7.589 events/10,000PDs during the first quarter of 2021 and a subsequent reduction in incidence to 5.295 events/10,000PDs in mid-2021 (Figure 3 and Supplementary Table S3).

## 4. Discussion

Our study provided accurate information on changes in antimicrobial consumption and AMR during the major waves of the COVID-19 pandemic. 

The strengths of our study were the extensive observation period, with seven years of temporal series data, accounting for almost two years of follow-up after the beginning of the COVID-19 pandemic, and a detailed analysis of all the main antibiotic classes collected for the whole hospital and the ICU. These characteristics could fill the knowledge gaps regarding the short-term and long-term changes in antibiotic consumption and antimicrobial resistance in the new epidemiological scenario caused by SARS-CoV-2.

Surprisingly, having extended the observation period to almost all of 2021, our results did not confirm the significant increase in antibiotic consumption or the HAI rate that was alarmingly reported by several observational studies at the beginning of the COVID-19 pandemic [6,15,17,23,24,25,26,27]. When comparing data from the first pandemic wave with those collected during the previous two years, Grau et al. observed an increase in the consumption of daptomycin, carbapenems, linezolid, ceftaroline, novel cephalosporin/β-lactamase inhibitors, and triazoles, especially in an ICU setting [25]. The same worrisome increase in antibacterial and antifungal consumption was also observed in a more extensive study that included 66 ICUs in Catalonia [26]. Similar results were also recently reported in The National Report on Antibiotics Use in Italy for the year 2020 published by the Italian Medicines Agency on 10 March 2022, with higher increases in carbapenems, macrolides, and 3GCs, especially in northern regions, which are the areas most affected by the pandemic. Of significant importance, the consumption of antibiotics that are active against MDROs increased to such an extent in 2020 that they accounted for nearly a quarter of the hospital’s antibiotic consumption [28].

We can assume that the 2020 global increase in AC was mainly due to the depletion of structural and human resources during the first pandemic wave, which also jeopardized the correct application of antimicrobial stewardship (AS) models. More importantly, empirical broad-spectrum antibiotic therapies were frequently administered to COVID-19 hospitalized patients, particularly critically ill patients, to treat suspected or confirmed bacterial co-infections and superinfections that likely occurred because of the severe clinical presentation and the need for oxygen support [2,3,24,29]. 

Our findings were not entirely in contrast with these results but, in addition, demonstrated that the increase in AC shown in the first COVID-19 wave was later controlled by restoring the ASP to pre-epidemic levels, particularly in the ICU relative to the rest of the hospital. Other recently published up-to-date reports that extended the follow-up period seemed to confirm our results [30]. As can be seen in Figure 1 and Figure 2, the consumption of 3GCs and macrolides increased in the early months of the pandemic, but their use gradually declined in 2021. Azithromycin was initially proposed not only for pneumonia co-infections but also as a primary treatment for COVID-19, alone or in combination with hydroxychloroquine, and was later discontinued at the end of 2020 due to a lack of benefit being demonstrated in a large randomized trial [31]. The inappropriate prescription of beta-lactam antibiotics and macrolides as empirical therapies during the COVID-19 pandemic was also common outside the hospital setting, especially in primary care [32,33,34,35]. Greater awareness of the low rate of bacterial co-infection in COVID-19 patients led to an important reduction in 3GC use. Moreover, while hospital overcrowding had compromised the correct implementation of AS, a decrease in AC could have been related to the dramatic decrease in surgical activities, including transplantation activities, and the discontinuation of all non-urgent routine healthcare activities. These encouraging results seemed to mitigate the potential long-term effect of initial inappropriate antibiotic prescriptions. 

Importantly, AS programs restarted after the first few years of the pandemic in many hospitals. Indeed, in our hospital, a widespread AS program based on prospective audits and feedback (PAF) that was implemented in 2015 and re-started after the first wave could have positively influenced the rapid restoration of appropriate prescriptions. This assumption is also supported by other colleagues who analyzed the effect of the pandemic on a persuasive educational antimicrobial stewardship program [36]. 

Considering microbiology, retrospective studies, together with global and national surveillance reports, indicated an increasing number of HAIs during the COVID-19 epidemic [15,17,37,38,39,40]. After analyzing HAIs from the National Healthcare Safety Network for 2019 and 2020 by acute-care hospitals, Weiner-Lastinger et al. reported a significant increase in the incidence of central-line-associated bloodstream infections, catheter-associated urinary tract infections, ventilator-associated events, and MRSA bacteremia in 2020 in the USA [15]. Similar results were also reported by Baker et al., who observed an increase in MDROs’ prevalence, which was strongly related to the COVID-19 burden [17,41]. Indeed, the number of clusters of nosocomial pathogens also increased during the COVID-19 waves [17,19,42]. Notably, cluster isolates accounted for 36% of the excess MDROs [19].

Such an increase may have been related to several factors: a longer hospital stay, the frequent need for invasive devices in particular mechanical ventilation, the inappropriate use of antibiotics, and mostly the patient-to-patient cross-transmission of healthcare-associated pathogens [17,39]. The increase in the incidence of Gram-positive MDROs, such as MRSA and vancomycin-resistant *E. faecium*, as our study also highlighted, are typically the result of clonal spread, which causes hospital outbreaks and seems to support this assumption [12,43,44]. 

Faced with an unprecedented threat and limited resources, healthcare systems adopted several countermeasures, including the deployment of medical staff primarily involved in the management of COVID-19, with the consequent relaxation of the regular IC programs being replaced by anti-COVID-19 measures. Although anti-COVID-19 measures were introduced to reinforce self-protection, they are not adequate to prevent MDRO cross-transmission. For example, double-gloving does not provide any additional protective benefit against SARS-CoV-2 over single-gloving and was shown to decrease hand hygiene. Glove disinfection in general may offer false hygiene assurance, leading to lower adhesion to the five moments of hand hygiene; moreover, disinfectants containing alcohol or bleach solution, which have been frequently used during the COVID-19 pandemic, promote glove breakage [45]. Therefore, the spread of nosocomial pathogens is strictly associated with a decline in hand hygiene compliance [19,40,43,46,47]. In addition, while the use of alcohol-based hand rubs could prevent the transmission of COVID-19, these solutions do not prevent CDI transmission, which requires strict adherence to handwashing with soap and water. Therefore, an increase in the trend of CDIs was registered in our study, though it did not reach statistical significance. When comparing the COVID-19 period versus the pre-pandemic era, the data are controversial, and most studies reported reduced or unchanged rates of CDIs [48,49,50]. According to a recent systematic review and meta-analysis of data collected between February 2020 and February 2021, CDI incidence rates ranged from 1.4 to 4.4 CDI cases per 10,000 patient-days [48]. The heterogeneity of these findings could also be explained by the difficulty in correctly diagnosing CDIs in the COVID-19 era [51,52]. The real impact of COVID-19 on the CDI burden is still unknown and requires further studies.

Finally, in our study, a statistically significant increase in BSIs due to carbapenem-susceptible *P. aeruginosa*, a well-known nosocomial pathogen, was observed. This finding could be related first to the extensive use of 3GCs, which was more evident during the first COVID-19 wave. Furthermore, the immunomodulation process that is typical of the SARS-CoV-2-infected patient related to the immunosuppressive effect mediated by the use of even high-dose steroid therapy may have played a crucial role. Another possible explanation relevant to our study could be related to the local ecology; indeed, *P*. *aeruginosa* in our hospital has been the most prevalent Gram-negative pathogen, while the prevalences of carbapenem-resistant *K. pneumoniae* and carbapenem-resistant *A. baumanii* (CRAB) were low in both the pre-COVID-19 and COVID-19 eras. This was likely due to the well-established implementation of IPC strategies, including universal screening for carbapenem-resistant organisms (CROs) and targeted interventions for each pathogen, such as our five-component bundle for permanently eliminating CRAB spreading [53]. We can speculate that thanks to these solid strategies, in contrast to other experiences [54,55,56,57,58], we did not observe an increased incidence of invasive CRO infections during the pandemic waves.

The increase in both the incidence of carbapenem-susceptible *P. aeruginosa* and MRSA highlighted during the first pandemic phase catalyzed the reinforcement of IPC-deficient policies, such as promoting the discontinuation of double-glove use, implementing distance learning courses on hand hygiene for healthcare personnel, including individual assessments for ICU health workers, recommending universal rectal screening for CROs, and promoting universal decolonization with mupirocin and chlorhexidine for MRSA-colonized patients. This educational activity was continued until IPC levels comparable with pre-pandemic levels were restored.

Our study had some limitations. First, being a single-center study, our results are not directly generalizable to other settings with different case-mix populations. Furthermore, our data referred to the entire hospital and it was not possible to distinguish between COVID and non-COVID areas. In addition, total isolates and ICU microbiological data were not included in the analysis due to the limited series. Finally, we did not analyze the confounding factors and possible mediators; therefore, we can only suggest that the lower AC was the most important cause of AMR incidence change during the COVID-19 pandemic. Further studies are necessary to improve the analysis methodology with updated data that are adjusted for the number of COVID bed-days and to perform an interventional analysis to understand the burden of each IPC and ASP.

## 5. Conclusions

While dealing with the major emergency of the COVID-19 pandemic, the spread of antimicrobial resistance, which was declared as one of the top ten global public health threats facing humanity by WHO in 2019, must not pass unnoticed.

Our findings demonstrated that the worrisome increase in antimicrobial consumption and antimicrobial resistance prevalence in the first COVID-19 wave was curbed in the subsequent period by quickly restoring infection prevention and control and antimicrobial stewardship programs to pre-epidemic levels. Without global efforts to re-implement these essential interventions, we might have to witness irreversible long-term consequences of COVID-19 on hospital antimicrobial resistance rates.

Our findings suggested that it is possible to mitigate the development of antimicrobial resistance through the periodic and radicalized application of antimicrobial stewardship and infection prevention and control policies. However, this outcome can only be achieved through already well-structured and efficient strategies, the implementation of which requires prior investments such that these strategies are already entrenched and thus can hold up even during periods of public health crises. This represents one of the most important lessons learned from this global pandemic. 

In the future, we need to think about investing to ensure the sustainability of these resources to deal with unpredictable situations, such as the COVID-19 pandemic, and to continue facing the longer-term global threat of antimicrobial resistance.

## Data Availability

The datasets used and/or analyzed during the current study are available from the corresponding author.

## Supplementary Materials

The following supporting information can be downloaded from https://www.mdpi.com/article/10.3390/antibiotics11060826/s1. Supplementary Table S1: Effect of the COVID-19 pandemic on antibiotic consumption at the University Hospital of Modena; Supplementary Table S2: Effect of the COVID-19 pandemic on antibiotic consumption in the ICUs of the University Hospital of Modena; Supplementary Table S3: Effect of the COVID-19 pandemic on the rate of bloodstream infections (BSIs) and *C. difficile* infections.

## Abbreviations

3GC	Third-generation cephalosporin
AC	Antimicrobial consumption
AMR	Antimicrobial resistance
AS	Antimicrobial stewardship
BSI	Bloodstream infection
CAESAR	Central Asian and European Surveillance of Antimicrobial Resistance
CDI	*Clostridioides difficile* infection
Cef R	Third-generation cephalosporin-resistant
Cef S	Third-generation cephalosporin-susceptible
CL	Change in level
COVID-19	Coronavirus disease 2019
CR	Carbapenem-resistant
CRAB	Carbapenem-resistant *Acinetobacter baumanii*
CR-GNB	Carbapenem-resistant Gram-negative bacteria
CRO	Carbapenem-resistant organism
CS	Carbapenem-susceptible
CT	Change in trend
DDD	Defined daily dose
EARS-Net	European Antimicrobial Resistance Surveillance Network
HAI	Healthcare-associated infection
ICU	Intensive care unit
ID	Incidence density
IPC	Infection prevention and control
MDROs	Multidrug-resistant organisms
MRSA	Methicillin-resistant *Staphylococcus aureus*
MSSA	Methicillin-susceptible *Staphylococcus aureus*
PAF	Prospective audit and feedback
PD	Patient-days
PPE	Personal protective equipment
SARS-CoV-2	Severe acute respiratory syndrome coronavirus 2
VAP	Ventilator-associated pneumonia
VR	Vancomycin-resistant
VS	Vancomycin-susceptible
