# Supplementary material for: Long-Term Impact of the COVID-19 Pandemic on In-Hospital Antibiotic Consumption and Antibiotic Resistance: A Time Series Analysis (2015–2021)
Source: Antibiotics (Basel). 2022 Jun 20;11(6):826. doi: 10.3390/antibiotics11060826 (PMC9219712; doi:10.3390/antibiotics11060826)
Supplement: Supplementary file 1 [file antibiotics-11-00826-s001.zip › _Long term impact Supplementary Tables.pdf]

Supplementary Table S1. Effect of the COVID-19 pandemic on antibiotic consumption at the University Hospital of Modena

Antibiotics	Parameter evaluated	Effect estimate	LCI	UCI	P value
**Carbapenems**	Change in trend	0.045	-0.015	0.10	0.14
	Change in level	0.197	-0.53	0.92	0.59
**Piperacillin/tazobactam**	Change in trend	-0.43	-0.69	-0.17	**0.001**
	Change in level	1.71	-1.23	4.64	0.25
**III Generation Cephalosporins**	Change in trend	0.014	-0.42	0.45	0.95
	Change in level	-2.03	-7.59	3.53	0.47
**Anti-Pseudomonal Cephalosporins**	Change in trend	-0.04	-0.16	0.07	0.45
	Change in level	-0.47	-1.80	0.85	0.48
**Amoxicillin/clavulanate**	Change in trend	-0.27	-0.49	-0.05	**0.018**
	Change in level	-2.20	-4.86	0.45	0.10
**Fluoroquinolones**	Change in trend	0.07	-0.14	0.28	0.52
	Change in level	-1.31	-3.81	1.19	0.30
**Glycopeptides**	Change in trend	-0.22	-0.31	-0.12	**<0.001**
	Change in level	0.50	-0.66	1.66	0.39
**Oxazolidinones**	Change in trend	-0.03	-0.09	0.02	0.28
	Change in level	0.11	-0.52	0.76	0.72
**Daptomycin**	Change in trend	-0.03	-0.09	0.03	0.28
	Change in level	0.45	-0.33	1.24	0.26
**Macrolides**	Change in trend	-0.30	-0.84	0.24	0.27
	Change in level	-0.63	-7.41	6.14	0.85
**Fosfomycin**	Change in trend	0.22	-0.01	0.44	0.06
	Change in level	-0.47	-3.20	2.25	0.73
**Other classes**	Change in trend	-0.25	-0.70	0.20	0.28
	Change in level	-1.03	-6.69	4.63	0.72
**All antibiotics**	Change in trend	-1.10	-2.06	-0.14	**0.025**
	Change in level	-3.03	-14.7	8.69	0.61

Footnotes: LCI Lower Confidence Interval; UCI: Upper Confidence Interval. Change in level positively affected by the COVID-19 pandemic highlighted in red; change in level negatively influenced by the COVID-19 pandemic highlighted in green

Supplementary Table S2. Effect of COVID-19 pandemic on antibiotic consumption in the ICUs of the University Hospital of Modena

Antibiotics	Parameter evaluated	Effect estimate	LCI	UCI	P value
**Carbapenems**	Change in trend	0.40	-0.21	1.01	0.19
	Change in level	-2.20	-9.49	5.08	0.55
**Piperacillin/tazobactam**	Change in trend	-0.59	-1.33	0.15	0.12
	Change in level	-3.51	-12.4	5.36	0.43
**III Generation Cephalosporins**	Change in trend	-0.75	-1.94	0.45	0.22
	Change in level	3.59	-10.99	18.18	0.62
**Anti-Pseudomonal Cephalosporins**	Change in trend	-0.08	-0.59	0.42	0.74
	Change in level	-1.28	-7.30	4.74	0.67
**Amoxicillin/clavulanate**	Change in trend	0.05	-0.40	0.49	0.84
	Change in level	-0.72	-6.03	4.58	0.79
**Fluoroquinolones**	Change in trend	-0.11	-0.61	0.39	0.66
	Change in level	-0.85	-6.93	5.23	0.78
**Glycopeptides**	Change in trend	-0.69	-1.45	0.07	0.07
	Change in level	-0.38	-9.49	8.72	0.93
**Oxazolidinones**	Change in trend	-0.21	-0.73	0.31	0.43
	Change in level	2.77	-3.42	8.97	0.37
**Daptomycin**	Change in trend	-0.67	-0.98	-0.37	**<0.001**
	Change in level	2.16	-1.50	5.82	0.24
**Tetracyclines**	Change in trend	-0.005	-0.68	0.67	0.99
	Change in level	-1.15	-9.17	6.86	0.77
**All antibiotics**	Change in trend	-4.47	-8.88	-0-06	**0.047**
	Change in level	17.27	-35.5	70.0	0.51

Footnotes: LCI Lower Confidence Interval; UCI: Upper Confidence Interval. Change in level positively affected by the COVID-19 pandemic highlighted in red; change in level negatively influenced by the COVID-19 pandemic highlighted in green

Supplementary Table S3. Effect of COVID-19 pandemic on the incidence of bloodstream infections (BSI) and C. difficile infections at the University Hospital of Modena.

	**Parameter evaluated**	**Effect estimate**	**LCI**	**UCI**	**P value**
Carbapenem-susceptible A. baumannii BSI	Change in trend	0.009	-0.013	0.031	0.406
	Change in level	-0.027	-0.250	0.196	0.812
Carbapenem-resistant A. baumannii BSI	Change in trend	0.020	-0.026	0.066	0.386
	Change in level	-0.173	-0.665	0.319	0.487
Carbapenem-susceptible P. aeruginosa BSI	Change in trend	-0.001	-0.128	0.126	0.991
	Change in level	1.477	0.130	2.824	**0.032**
Carbapenem-resistant P. aeruginosa BSI	Change in trend	-0.009	-0.051	0.033	0.675
	Change in level	0.133	-0.319	0.5985	0.559
Carbapenem-susceptible K. pneumoniae BSI	Change in trend	-0.022	-0.169	0.125	0.767
	Change in level	0.322	-1.230	1.874	0.681
Carbapenem-resistant K. pneumoniae BSI	Change in trend	-0.023	-0.071	0.025	0.342
	Change in level	0.344	-0.162	0.850	0.179
III generation Cephalosporin-susceptible E. coli BSI	Change in trend	0.162	-0.137	0.461	0.284
	Change in level	-0.823	-3.973	2.326	0.604
III generation Cephalosporin-resistant E. coli BSI	Change in trend	0.036	-0.097	0.169	0.592
	Change in level	-0.487	-1.897	0.923	0.493
Methicillin-susceptible S. aureus BSI	Change in trend	-0.017	-0.184	0.150	0.841
	Change in level	-1.482	-0.295	3.259	0.101
Methicillin-resistant S. aureus BSI	Change in trend	-0.078	-0.150	-0.006	**0.034**
	Change in level	0.722	-0.039	1.482	0.062
Vancomycin-susceptible E. faecium BSI	Change in trend	-0.062	-0.180	0.057	0.269
	Change in level	1.006	-0.245	2.257	0.092
Vancomycin -resistant E. faecium BSI	Change in trend	0.007	-0.053	0.066	0.803
	Change in level	-0.097	-0.744	0.549	0.751
C. difficile Infections	Change in trend	1.43	-0.002	2.86	0.051
	Change in level	-0.94	-3.97	2.09	0.52

Footnotes: LCI Lower Confidence Interval; UCI: Upper Confidence Interval. Change in level positively affected by the COVID-19 pandemic highlighted in red; change in level negatively influenced by the COVID-19 pandemic highlighted in green
